# Supplementary material for: Synaptotagmin 1 Suppresses Colorectal Cancer Metastasis by Inhibiting ERK/MAPK Signaling-Mediated Tumor Cell Pseudopodial Formation and Migration
Source: Cancers (Basel). 2023 Nov 3;15(21):5282. doi: 10.3390/cancers15215282 (PMC10649299; doi:10.3390/cancers15215282)
Supplement: Supplementary file 1 [file cancers-15-05282-s001.zip › cancers-2644128-supplementary.pdf]

cancers MDPI

# Supplementary Information

*Article*

# Synaptotagmin 1 Suppresses Colorectal Cancer Metastasis by Inhibiting ERK/MAPK Signaling-Mediated Tumor Cell Pseudopodial Formation and Migration

**Jian-Yun Shi [1,†], Wen-Jing Li [1,†], Zhen-Hua Jia [1], Ying Peng [1], Jia-Yi Hou [2], Ning Li [3], Rui-Juan Meng [4], Wei Fu [4], Yan-Lin Feng [1], Li-Fei Wu [1], Lan Zhou [1], De-Ping Wang [1], Jing Shen [1], Jia-Song Chang [1], Yan-Qiang Wang [5,*] and Ji-Min Cao [1,*]**

1 Key Laboratory of Cellular Physiology at Shanxi Medical University, Ministry of Education, and the Department of Physiology, Shanxi Medical University, Taiyuan 030606, China; shijianyun@sxmu.edu.cn (J.-Y.S.); liwenjing2@sxmu.edu.cn (W.-J.L.); jiazhenhua@sxmu.edu.cn (Z.-H.J.); pengying2@sxmu.edu.cn (Y.P.); feng@sxmu.edu.cn (Y.-L.F.); lifeiwu891002@sxmu.edu.cn (L.-F.W.); zhoulan@sxmu.edu.cn (L.Z.); wangdeping@sxmu.edu.cn (D.-P.W.); shenjing11@sxmu.edu.cn (J.S.); changjiasong@sxmu.edu.cn (J.-S.C.)
2 Department of Clinical Laboratory, Shanxi Provincial Academy of Traditional Chinese Medicine, Taiyuan 030071, China; youhoujiayi0103@126.com
3 Department of Gastrointestinal and Pancreatic Surgery & Hernia and Abdominal Surgery, Shanxi Provincial People's Hospital, Taiyuan 030045, China; lining205310@126.com
4 Department of Radiology, The First Hospital of Shanxi Medical University, Shanxi Medical University, Taiyuan 030606, China; mrj0601@163.com (R.-J.M.); fuwei001@163.com (W.F.)
5 Translational Medicine Research Center, Shanxi Medical University, Taiyuan 030606, China
* Correspondence: yqwang15@sxmu.edu.cn (Y.-Q.W.); caojimin@sxmu.edu.cn (J.-M.C.)
† These authors contributed equally to this work.

**Table S1.** Results of Cox regression analysis.

| Characteristics | Total(N) | Univariate analysis | | Multivariate analysis | |
|---|---|---|---|---|---|
| | | Hazard ratio (95% CI) | P value | Hazard ratio (95% CI) | P value |
| T stage | 640 | | | | |
| T1&T2 | 131 | Reference | | | |
| T3&T4 | 509 | 2.468 (1.327-4.589) | **0.004** | 1.926 (0.810-4.577) | 0.138 |
| N stage | 520 | | | | |
| N0 | 367 | Reference | | | |
| N1 | 153 | 1.803 (1.149-2.829) | **0.010** | 0.768 (0.368-1.604) | 0.482 |
| M stage | 563 | | | | |
| M0 | 474 | Reference | | | |
| M1 | 89 | 3.989 (2.684-5.929) | **<0.001** | 5.384 (2.477-11.699) | **<0.001** |
| Age | 643 | | | | |
| <=65 | 276 | Reference | | | |
| >65 | 367 | 1.939 (1.320-2.849) | **<0.001** | 2.499 (1.355-4.607) | **0.003** |
| Gender | 643 | | | | |
| Female | 301 | Reference | | | |
| Male | 342 | 1.054 (0.744-1.491) | 0.769 | | |
| Lymphatic invasion | 581 | | | | |
| No | 349 | Reference | | | |
| Yes | 232 | 2.144 (1.476-3.114) | **<0.001** | 1.763 (1.010-3.076) | **0.046** |
| SYT1 | 643 | | | | |
| Low | 321 | Reference | | | |
| High | 322 | 1.487 (1.045-2.115) | **0.027** | 1.940 (1.112-3.384) | **0.020** |

**Table S2.** Sources of antibodies used in this study.

| Antibodies | Manufacturer |
|---|---|
| SYT1 | CST, 14558S, Rabbit, USA |
| GAPDH | Beyotime, AF0006, Mouse, CHN |
| Beta-Tubulin | Affinity, T0023, Mouse, USA |
| Slug | CST, 9585T, Rabbit, USA |
| Vimentin | CST, 5741T, Rabbit, USA |
| ERK1/2 | CST, 4695T, Rabbit, USA |
| p-ERK1/2 | CST, 4370T, Rabbit, USA |
| Ki67 | CST, 9449S, Mouse, USA |
| PCNA | CST, 2586T, Mouse, USA |

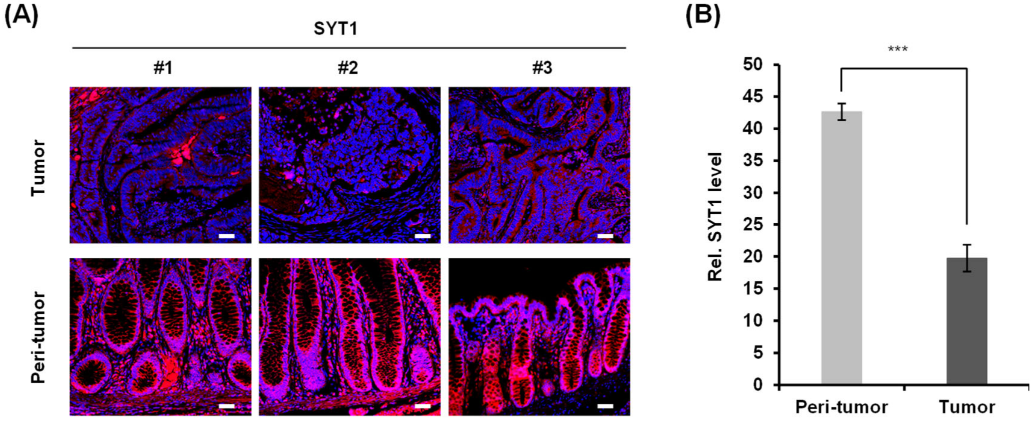


**Figure S1.** SYT1 suppressed the progression of CRC. (A) Three representative images of immunofluorescence analysis of SYT1 protein in three pairs of CRC tissues and peri-tumor. (B) Statistical analysis of all 15 pairs of samples was calculated. * $p < 0.05$, ** $p < 0.01$, *** $p < 0.001$. Scale bar, 50 µm.

Figure 1

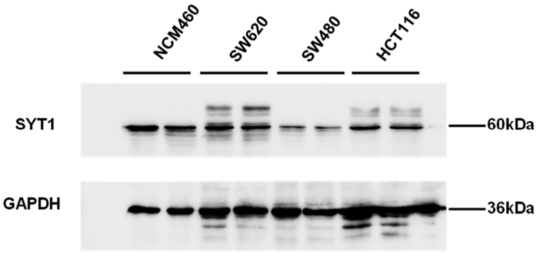


Figure 2

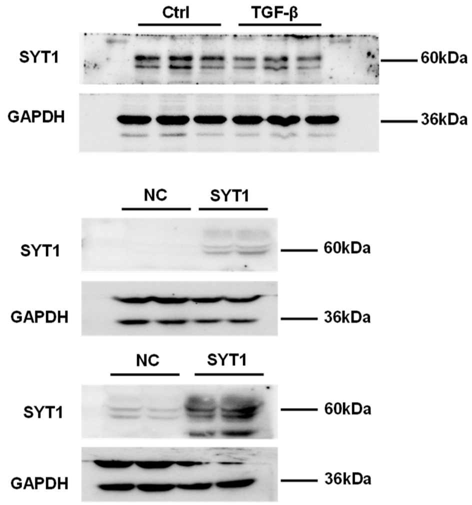


Figure 5

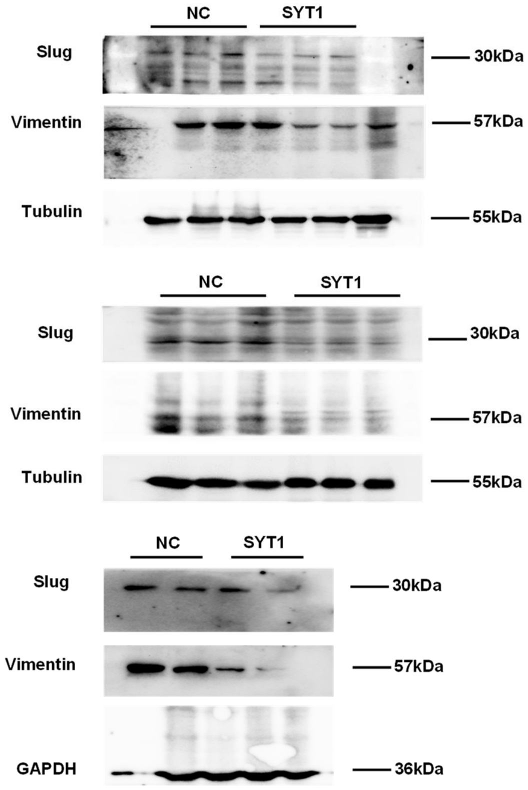


Figure 6

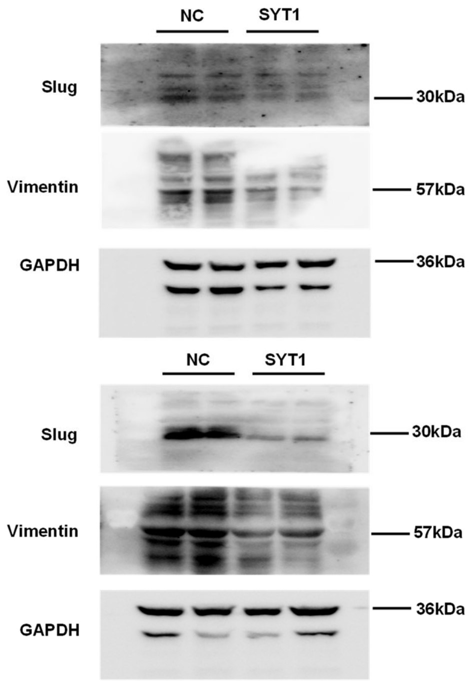

Figure 7

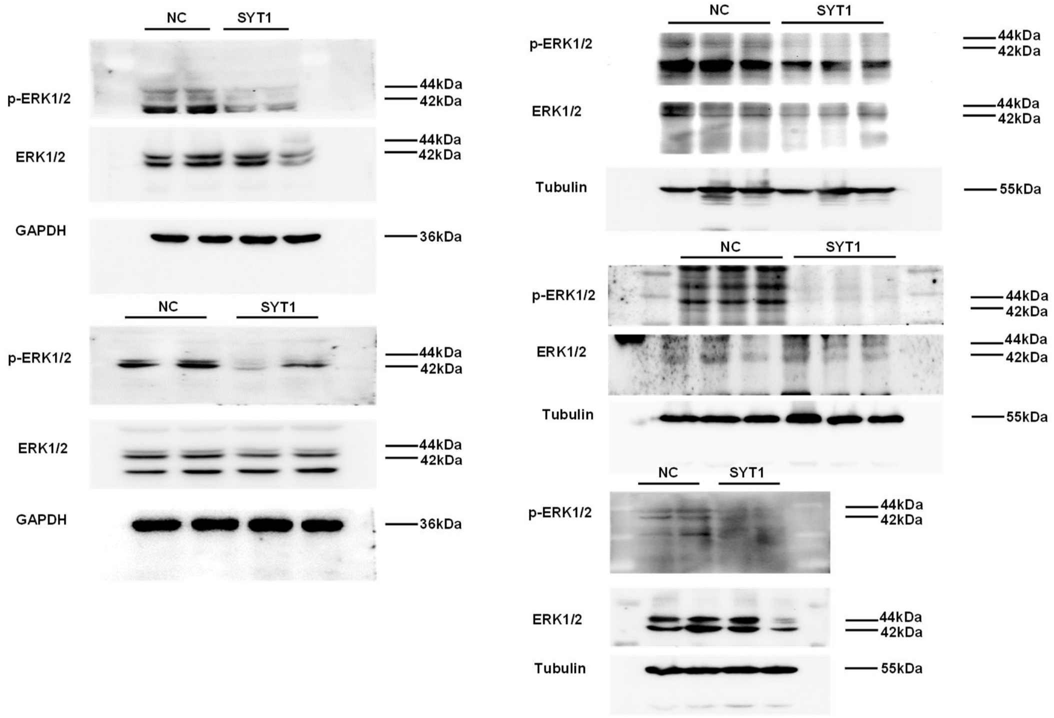


Figure 8

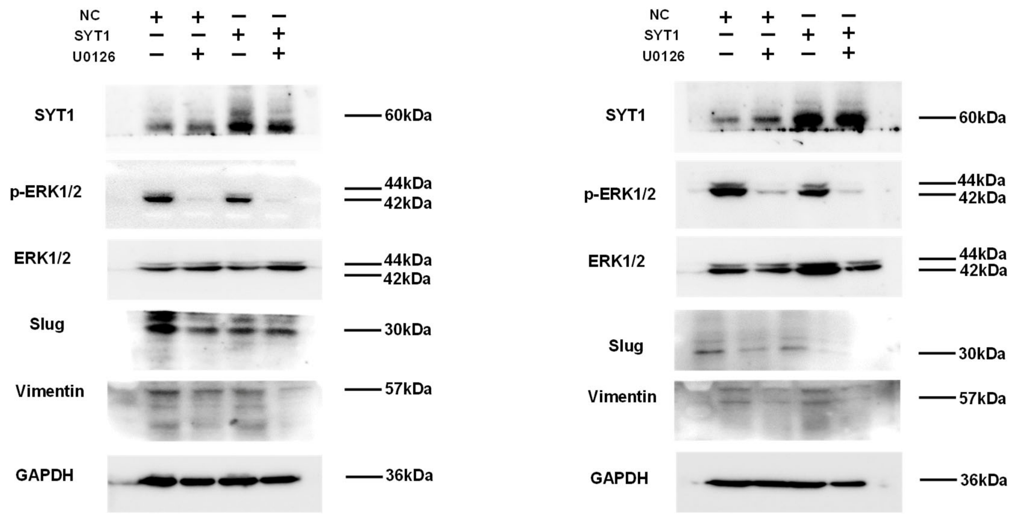


**Figure S2.** Original blots for Figures 1-8.
